# The Structure of Performance of a Sport Rock Climber

**DOI:** 10.2478/hukin-2013-0011

**Published:** 2013-03-28

**Authors:** Artur Magiera, Robert Roczniok, Adam Maszczyk, Miłosz Czuba, Joanna Kantyka, Piotr Kurek

**Affiliations:** 1 Academy of Physical Education, Department of Tourism and Sport Management, Katowice, Poland.; 2 Academy of Physical Education, Department of Sports Theory, Katowice, Poland.; 3 Department of Physiotherapy, University of Technology, Opole, Poland.

**Keywords:** sport climbing, canonical analysis, structure of performance

## Abstract

This study is a contribution to the discussion about the structure of performance of sport rock climbers. Because of the complex and multifaceted nature of this sport, multivariate statistics were applied in the study. The subjects included thirty experienced sport climbers. Forty three variables were scrutinised, namely somatic characteristics, specific physical fitness, coordination abilities, aerobic and anaerobic power, technical and tactical skills, mental characteristics, as well as 2 variables describing the climber’s performance in the OS (Max OS) and RP style (Max RP). The results show that for training effectiveness of advanced climbers to be thoroughly analysed and examined, tests assessing their physical, technical and mental characteristics are necessary. The three sets of variables used in this study explained the structure of performance similarly, but not identically (in 38, 33 and 25%, respectively). They were also complementary to around 30% of the variance. The overall performance capacity of a sport rock climber (Max OS and Max RP) was also evaluated in the study. The canonical weights of the dominant first canonical root were 0.554 and 0.512 for Max OS and Max RP, respectively. Despite the differences between the two styles of climbing, seven variables – the maximal relative strength of the fingers (canonical weight = 0.490), mental endurance (one of scales : The Formal Characteristics of Behaviour–Temperament Inventory (FCB–TI; Strelau and Zawadzki, 1995)) (−0.410), climbing technique (0.370), isometric endurance of the fingers (0.340), the number of errors in the complex reaction time test (−0.319), the ape index (−0.319) and oxygen uptake during arm work at the anaerobic threshold (0.254) were found to explain 77% of performance capacity common to the two styles.

## Introduction

Researchers have been attracted to rock climbing since late 1970s, partly because of its increasing popularity and also due to the rising interest in making it one of the Olympic sport disciplines. Recently, research has concentrated on sport climbing where climbers are protected against falling from a height by permanent protection points installed along climbing routes. At present, these precautions are typical of events involving artificial climbing walls, as well as being frequently used during outdoor climbing events, mostly on rocks rising several tens of meters high.

The performance of sport rock climbers is judged by their ability to complete a route presenting a certain level (grade) of technical difficulty in one of three climbing styles. The most popular styles are defined based on whether climbers set out to complete a route without any previous knowledge of it (*on sight – OS)*, or whether they successfully reach the endpoint without falling off after gaining some experience of the route during earlier trials *(red point – RP).*

Although the number of studies dealing with this sport has grown, the results are conflicting (Espana-Romero, 2009; Giles et al., 2006; Watts, 2004), probably because of the complex and multifaceted nature of climbing. These circumstances provided grounds for attempting to identify the structure of climber’s performance by means of canonical analysis, a tool of multivariate statistics.

Previous studies on sport climbing (Mermier et al., 2000; Giles et al., 2006) used regression analysis to find correlations between one dependent variable Y and a set of independent variables {X1,…Xn}. This approach has been found insufficient, though, when the object of analysis is a set of dependent variables {Y1,…Yn}. The canonical analysis is used in such cases and it seeks correlations between two sets (vectors) of variables. Basically, canonical analysis aims:

▪ to find uncorrelated canonical variables that explain an increasingly large amount of variance in two sets,
▪ to calculate canonical weights describing each variable’s „pure” contribution to the canonical variable,
▪ to calculate factor loadings that determine each variable’s correlation with the canonical variable,
▪ to calculate the extracted variance and then redundancy showing the average amount of variance in one data set that the canonical variable explains through the variables of the second set.

Although used as a means of studying other sport disciplines (Babić et al., 2007; Blažević, 2009; Malacko, 2010), canonical analysis has never been applied to explore the structure of performance in sport rock climbing. In this study, it was chosen to answer the following research questions:

▪ which variables explain the climber’s performance in sport rock climbing to the highest degree, regardless of the climbing style?
▪ how do the sets of various mental, technical and physical characteristics affect two dependent variables: best performance in the OS style and best performance in the RP style?
▪ how are the vectors of the three sets of characteristics correlated?

## Material and Methods

Thirty Polish advanced male climbers (average performance in the OS style: 7b+ (7a - 8a); average performance in the RP style: 8a (7b+ - 8b+/8c) volunteered to participate in this study. This group was analysed previously in research of Magiera and Rygula (2007). Their age was 27 ± 5.45 years, the climbing experience 8.4 ± 3.46 years and the weekly training time 10 ± 3.59 hours. The methods for data collection were direct observation. Physiological, motor and psychological tests were carried out under standard conditions. Most of the tests were dedicated to sport climbing, climber’s experience and age.

The variables included 45 somatic and mental characteristics, specific physical fitness, coordination abilities, aerobic and anaerobic power, technical and tactical skills. Self-reported onsight (Max OS) and redpiont (Max RP) climbing performance were determined as the most difficult. To ensure that the route grading systems were comparable and to make them useful for mathematical analyses, a decimal scale (Köstermeyer, 2000) and a conversion table were used. The description of measuring instruments has been omitted. Their detailed description can be found in the study of Magiera (2006).

The first step in the subsequent statistical analysis was the calculation of basic statistical measures, such as an arithmetic average (X), standard deviation (S), coefficient of variation (V), coefficient of asymmetry (A_s_), and coefficient of kurtosis (K_u_-3) (Table 1). Further mathematical and statistical analysis utilised a multivariate exploration technique – canonical analysis. The statistically significant correlations between two different sets of variables were sought using: λ – significance of the square of canonical correlation, Rc – the canonical correlation value, Rc^2^ – the values of the squares of canonical correlations, χ^2^-chi-square values of Bartlett’s test, and p – statistical significance at < 0.05 (Malacko, 2010).

## Results

To be able to answer the question „Which characteristics explain the climber’s performance in sport rock climbing to the highest degree, regardless of the climbing style?” two sets of variables were compared:

▪ dependent variables – *Max OS* and *Max RP*
▪ independent variables – common characteristics obtained from two regression equations M*ax OS and Max RP*.

The findings from the analysis of the two sets of variables are shown in Table 2.

The next step of the research involved the calculation of the values of the variables and their canonical correlations and testing them for significance. Two canonical variables were calculated, whose correlations (Rc) for the first and second variable were 0.94 and 0.54, respectively. Both correlations were statistically significant (p<0.05), thus showing that the model described both data sets well. With the calculation of the variance and redundancy values it was possible to identify the amounts of variance explained by particular canonical variables. The first root extracted from the performance indicators (*Max OS* and *Max RP*) around 88% of the variance, while the second one only 12%. The redundancy value for the first root indicated that the independent variables (set II) explained 77% of the variance in climbing performance (p<0.05). Because the first canonical variable explained a much larger amount of the variance (81%) than the total redundancy value, it was concluded that it described the analysed phenomenon well. Hence further analysis concentrated on this variable.

By looking at the factor structure of the above sets of variables the correlations between the canonical roots and the variables in the set could be identified. The factor loadings of the first root were very similar (*Max OS*: −0.94; *Max RP*: −0.93), showing that both the results were equivalent and that neither of the climbing styles tended to dominate. The factor loadings of the first root for the independent variables were the following: Ape index: 0.303, CTR-errors: 0.445, Finger strength: −0.554, E70%z10/10: −0.035, VO_2_AT_Arm_: −0.558, TEMP-ME: 0.256, Technique: −0,622.

Therefore, the first canonical variable was represented by two equations:

$${\text{U}}_{1}=0.554\times \text{Max}\;\text{OS}+0.512\times \text{Max}\;\text{RP}$$

$${\text{V}}_{1}=-0.319\times \text{Ape}\;\text{index}-0.319\times \text{CTR-errors}+0.490\times \text{Finger}\;\text{strength}+0.340\times \text{E}70\%\text{z}10/10+0.254\times {\text{VO}}_{2}{\text{AT}}_{\text{Arm}}-0.410\times \text{TEMP-ME}+0.370\times \text{Technique}$$

The canonical analysis was also useful in determining how a set of different characteristics (technical, physical and mental) affected two dependent variables *Max OS and Max RP* used in the study, thus giving the answer to the second research question.

To make comparisons more efficient, eight characteristics were selected from each of the three sets of climbers’ mental, technical and physical attributes (Table 3). The first and most significant canonical correlations in the new sets of mental characteristics (personality traits, temperament, locus of control and tactics), technical characteristics (coordination and technique) and physical characteristics (somatic, flexibility, physical fitness and efficiency) were high, the canonical R being 0.82, 0.81 and 0.79, respectively. All correlations were statistically significant (p<0.001). The total redundancy values for the three sets interpreted as average percentages of the variance in one set of variables that all canonical variables explained based on another set were differentiated. This means that in analysing climber’s performance (the *Max OS* and *Max RP* set) eight mental characteristics explained 41% of the variance, eight technical characteristics – 53%, and eight physical characteristics – 62%.

The canonical analysis helped answer the third question too. The first to be analysed were the sets of somatic and physical fitness characteristics and that of coordination and technique (Table 4, columns 2 and 3). The total canonical R was high (0.82) and statistically significant (p<0.001). The canonical roots in the right set (the vectors of physical characteristics) explained almost 32% of the variance in the left set of variables (technical characteristics). Reversely, the first set explained 29% of the variance. The results obtained from comparing the characteristics of personality, temperament, locus of control and tactics with the somatic and physical fitness characteristics (Table 4, columns 4 and 5) showed that the right set (mental characteristics) explained almost 30% of the variance in the left set (physical characteristics). In the reverse situation, the rate of the explained variance declined to 25%. The total canonical R was both high (0.83) and statistically very significant (p<0.001). The sets of mental and technical characteristics were compared last (Tables 4, columns 6 and 7). The total canonical R was similar to its values determined from the previous analyses (0.82) and also statistically very significant (p<0.001). The canonical roots of both the right set and the left set explained a similar amount of the variance – 38%.

## Discussion

The available studies determine climber’s performance from questionnaire surveys (where the respondents are asked to state the most difficult route they have completed in the OS or RP style) (Booth et al., 1999; Espana-Romero et al., 2009; Ferguson and Brown, 1997; Grant et al., 2003; Müller and Held, 1992; Sheel et al., 2003), based on the score in a climbing test carried out in a setting made to resemble a lead climbing event (Mermier et al., 2000), or by calculating an Athlete Development Indicator (ADI) by means of the Hellwig’s algorithm (Magiera and Ryguła, 2007). Whatever the approach, the test batteries invariably address one, special type of performance achievable in different climbing styles or in different climbing settings (indoor or outdoor).

The approach taken in this study allowed to look at climbing performance from a somewhat broader perspective. Canonical analysis provided *Max OS* and *Max RP* performances which were taken to represent the overall performance capacity of a sport rock climber. The analysis found the following variables to be significant in the equation of the dominant first root: maximal relative strength of the fingers (*Finger strength:* 0.490), mental endurance (*TEMP-ME:* −0.410) and technique (*Technique*: 0.370), followed by isometric endurance of the fingers (*E70%z10/10:* 0,340), the number of errors in the complex reaction time test (*CRT-errors*: −0,319), ape index (−0,319) and oxygen uptake during arm work at the anaerobic threshold (*VO_2_AT_Arm_*: 0,254). These seven characteristics described the climber’s overall performance capacity well, explaining 77% of its variance. This may mean that despite their distinctive requirements, climbing styles are of little effect on performance unlike climber’s general abilities. Other available studies only deal with some of the model variables.

Notwithstanding the aforementioned disagreement over what determines sport climber’s performance, many studies treat finger strength as a prerequisite for its high level (Espana-Romero et al., 2009; Giles et al., 2006; MacLeod et al., 2006; Quaine and Vigouroux, 2004; Watts, 2004). This study confirmed this view. According to the canonical values, this variable (*Finger strength*) was the most significant. The greater maximal strength of the four fingers (without the thumb), particularly in relation to climber’s body mass, the better performance in climbing.

Earlier studies tended to give more attention to climber’s endurance. This ability has been assessed with many different tools, but recently tests evaluating the isometric endurance of the finger flexors have come to the fore (Ferguson and Brown, 1997; MacLeod et al., 2006; Quaine et al., 2003), as well as tests utilising climbing ergometers (Espana-Romero et al., 2009; Köstermeyer, 2000). The results of the first type of tests have showed that better forearm vascular capacity increases climber’s performance during the workload-relaxation sequence by allowing more blood to be supplied to muscles between contractions. In the second case, the climbing time (Espana-Romero et al., 2009) or the distance completed in a test with a climbing ergometer (Köstermeyer, 2000) have been strongly correlated with performance, particularly in experienced climbers. Maximal oxygen uptake in the incremental test to exhaustion did not differentiate the subjects (Espana-Romero et al., 2009), but the distance completed in a state of functional equilibrium has been found to significantly affect the endurance test results (Köstermeyer, 2000). These findings are confirmed by variables *E70%z10/10* and *VO_2_AT_Arm_* used in this study.

The role that the ‘ape index’ variable (the arm span to height ratio) plays in the model has not been fully explained. Inversely proportional effect of this variable on performance may be controversial. The authors assume that the arm span which does not differentiate most climbers in most cases (Espana-Romero et al., 2009) is less important than having a slimmer body (i.e. a smaller shoulder width). This opinion requires further investigations.

Canonical analysis was used in this study also to identify the structure of performance of sport rock climbers with respect to their various technical, physical and mental characteristics. Previous studies sought relationships between performance and particular somatic, physical fitness, physiological or mental characteristics. Interdisciplinary papers analysing climbers from many angles are not available. An exception is the studies carried out by Mermier et al. (2000) and Magiera and Ryguła (2007).

In the Mermier et al.’s study (2000) the principal component analysis (PCA) allowed extracting three components which were called „a training component” (the strength of the arms and legs and of the full-hand grip, the anaerobic power of the upper and lower body, arm endurance, % fat, climbing performance), „an anthropometric component” (body mass and height, the length of the lower extremities, arm span, ape index), and „a flexibility component” (the hip-joint range of motion). The authors have proven that being successful in climbing depends on the interaction of many factors rather than on a single factor, as suggested before. Multiple regression of the relationships between the three components and the subjects’ overall scores in two climbing trials showed that the components explained 58.9% (training), 0.3% (anthropometric) and 1.8% (flexibility) of the total variance in performance. The authors themselves suggested that more in-depth studies allowing also for mental and technical characteristics and technical and tactical skills were necessary to explain the remaining 34% of the variance in climbing performance.

The primary research purpose of the Magiera and Ryguła study (2007) was to build a biometric model describing the best performance of male climbers in the OS style based on an Athlete Development Indicator (ADI). It was almost completely (R^2^=0.93) explained by 9 variables providing the best description of this phenomenon: technical skills, oxygen uptake during arm work at the anaerobic threshold, maximal relative strength of the fingers, locus of control, psychotism, strength endurance*,* ape index, the number of errors in the complex reaction time test and the range of motion during hip flexion.

**Figure 1:**
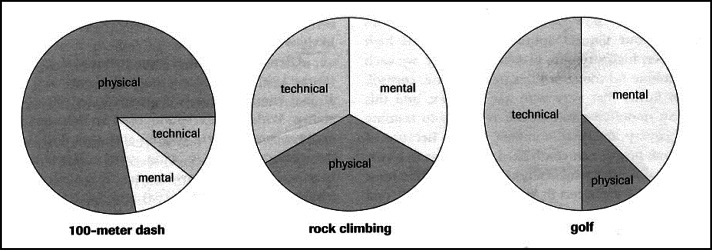
The relative requirements of different sports (Hörst, 2003)

Scientists studying this sport discipline have also made attempts to assess how particular attributes of climbers contribute to their performance. Hörst (2003), who is an author of many popular climbing handbooks, views rock climbing as a unique sport where the athlete is required to demonstrate almost a complete balance of mental characteristics, technical skills and physical abilities. He contrasts it with sports where performance is mainly determined by physical characteristics (100m sprint) or technical skills (golf) (Figure 1). Unfortunately, it is only a subjective opinion of the author, without any scientific background.

**Figure 2:**
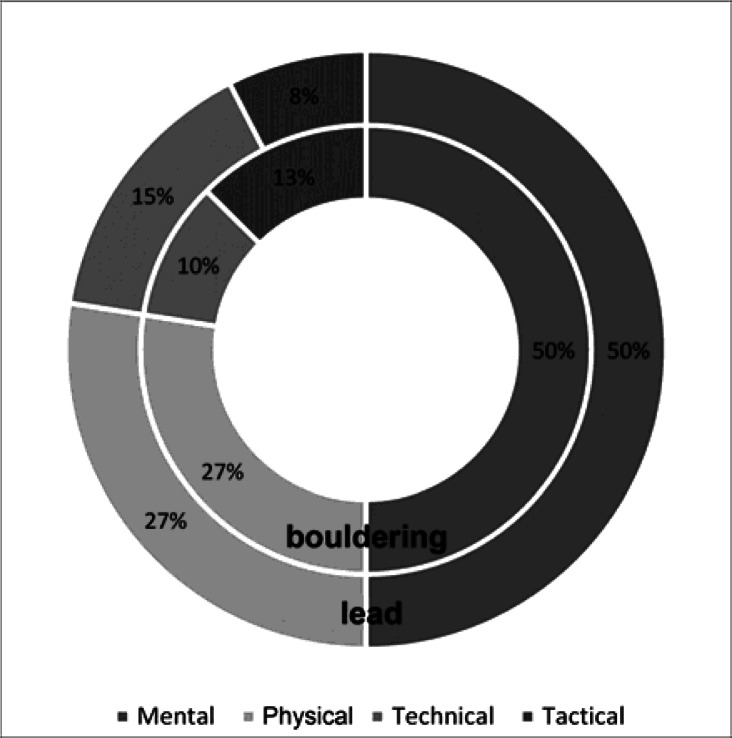
The structure of sport climber’s performance in the lead and bouldering events (Guidi, 2002)

Guidi has a different opinion in regard to this topic. In his report published on the official website of the FFME (Fédération Française de la Montagne et de l'Escalade) Guidi presented the findings of an expert commission consisting of the FFME coaches (Guidi, 2002). Among other things, he analysed the structure of climbers’ performance in the lead and bouldering events (Figure 2). According to Guidi, the key factors determining performance in the first event were mental characteristics (50%), then physical (27%), tactical (15%) and technical (8%) ones.

**Figure 3:**
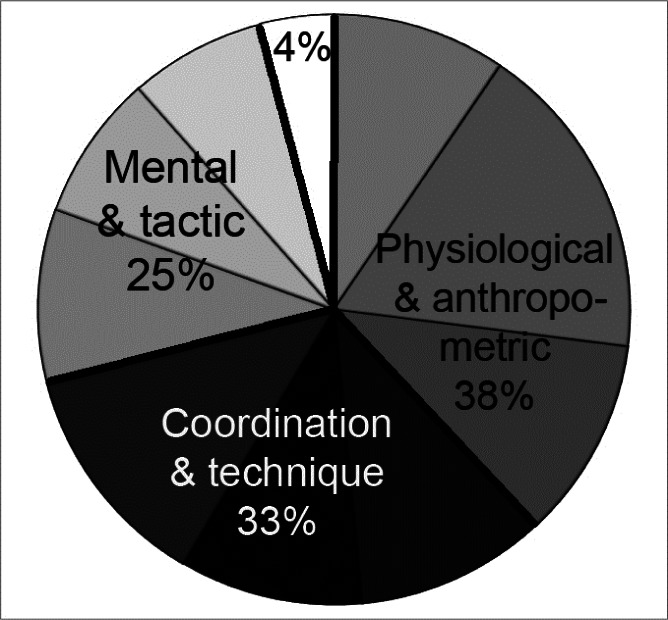
Percentage contributions and the complementarity of different sets of characteristics explaining climber’s overall performance capacity (Max OS and Max RP)

The findings of this study where the issue of climber’s performance has been given comprehensive treatment allowed empirical verification of the above opinions. According to the results of the canonical analysis (Table 3) and their totals (Figure 3), three sets of characteristics, each having 8 selected variables, explained climbers’ overall performance capacity in 96% (*Max OS* and *Max RP*). The chart below tends to support the Hörst’s opinion (2003) that rock climbing requires harmoniously developed physical fitness, technical and tactical skills, as well as mental preparation. The percentage contributions of particular sets of variables to explaining performance were similar, but not equal. The characteristics of physical fitness (*Finger strength, E70%z10/10, Arm strenght*), body efficiency (*VO_2_AT_Arm_*) and anthropometric (*Body mass, Ape index, FM%, Hip flexion*) explained the most – 38%, while mental characteristics were found to be the least significant in this respect (25%). The present-day sport climbing is safer for contestants (owing to permanent protection points, strong ropes, etc.). Climbers are viewed today as gymnasts exercising on the rock rather than people risking their lives en route to the top. This safety and the outstanding experience of the examined climbers not only seem to explain the relatively low share of mental attributes in the structure of their performance, but also highlight the prominence of the physical aspects of their training.

This study has shown that sport climbing performance is determined by different sets of morphofunctional characteristics. Keeping the sets apart has only a theoretical advantage, because they are in fact complementary and overlap (Figure 3). Climbers, particularly the less trained ones, frequently utilise this interaction to compensate for their deficiencies with better developed skills and abilities. The canonical analysis may be a measure to find out whether variables in one set may serve as predictors of the values of the variables in another. All three sets of characteristics (physical, mental and technical) used in this study explained the variance similarly (in around 30%), but the strongest relationship was found between the set containing selected characteristics of personality, temperament, locus of control and tactics, and the set with coordination abilities and technique (38 %). This seems to explain why the two groups of characteristics have a similar informative value. The climbers’ physical characteristics were explained least effectively by their mental attributes (25%), which reveals a relatively weaker relationship between the results of selected mental tests and the somatic, physical fitness, aerobic and anaerobic power of the climbers

This study focused on advanced male climbers taking part in rock climbing events. For different sex and experience of the subjects, type and setting of the events (indoor or outdoor), the results may be different.

## Conclusions

A thorough study of training efficiency of advanced sport climbers involves testing of their physical, technical and mental characteristics. The three sets of characteristics used in this study explained the structure of climbing performance to a similar, but unequal degree, i.e. in 38, 33 and 25%, respectively. The sets were also found to be complementary to around 30% of the variance. The study determined also the overall performance capacity of outdoor climbers. Although the OS and RP climbing styles pose different requirements, seven variables explained 77% of climber’s overall performance capacity common to the two styles. An insight into its structure was enabled by the canonical analysis, a tool of multivariate statistics.

## Figures and Tables

**Table 1:** Descriptive statistics

**N**		**Variables**		**X**	**S**	**V**	**A_S_**	**K_u_-3**
**1.**	**Max OS**	Best performance in OS style	n	8,68	0,53	6,08	−0,05	−1,39
**2.**	**Max RP**	Best performance in RP style	n	9,55	0,55	5,80	0,22	−1,12
**3.**	**Mass**	Body mass	kg	68,85	5,02	7,30	−0,73	1,22
**4.**	**Height**	Height	cm	177,90	5,59	3,14	0,04	−0,94
**5.**	**Arm span**	Arm span	cm	180,09	7,02	3,90	−0,10	−1,15
**6.**	**Ape index**	Ape index: arm span/height	cm/cm	1,01	0,02	2,33	0,63	0,38
**7.**	**FM%**	% of fat tissue	%	10,42	3,28	31,47	0,27	−0,50
**8.**	**MM%**	% of muscle tissue	%	63,77	8,30	13,01	0,31	0,40
**9.**	**BMI**	Body Mass Index	kg/m^2^	21,82	1,70	7,78	−0,03	−0,30
**10.**	**BCMI**	Body Cell Mass Index	kg/m^2^	11,35	2,03	17,86	0,19	−0,24
**11.**	**Hip flexion**	Range of motion of hip flexion	st.	118,67	9,95	8,38	0,09	−1,42
**12.**	**Hip abduct**	Range of motion of hip abduction	st.	51,30	6,95	13,55	−0,19	0,29
**13.**	**Froggies**	Flexibility of hips in “froggies”	cm	6,11	5,10	83,41	0,23	−0,24
**14.**	**CRT-errors**	Complex reaction time – number of errors	n	5,87	2,79	47,54	−0,11	−0,78
**15.**	**Stereometry**	Stereometry	mm	14,33	10,09	70,36	1,05	0,08
**16.**	**Balance-inst**	State of balance – instability	st./s	260,98	54,45	20,86	−1,64	2,95
**17.**	**Balance-lc**	State of balance – locus of control	n	81,80	8,80	10,76	0,13	−0,84
**18.**	**Adapt-error**	Motor adaptation – error	S*T	168,13	55,77	33,17	0,83	−0,14
**19.**	**Adapt-rate**	Motor adaptation – adaptation rate	s	0,84	0,25	30,09	1,53	2,74
**20.**	**Different**	Differentiation	%	87,50	11,53	13,18	−1,08	0,93
**21.**	**Finger strength**	Maximal finger strength	kg/kg	0,55	0,06	11,39	−0,33	−0,37
**22.**	**E70%z10/10**	Finger endurance 10/10s 70%Fmax	s	358,80	198,67	55,37	1,57	2,02
**23.**	**Arm strength**	Arm strength	kg/kg	1,64	0,12	7,44	0,16	−0,63
**24.**	**Arm endurance**	Arm endurance	s	67,43	13,68	20,28	0,03	−0,97
**25.**	**W30s-Wtotal**	Total work of the upper body - W30s	J/kg	157,37	11,50	7,31	−0,93	1,16
**26.**	**W30s-Pmax**	Maximal power of the upper body - W30s	W/kg	6,43	0,38	5,92	−0,46	0,41
**27.**	**W30s-Fatigue**	Fatigue index - W30s	%	17,90	3,10	17,29	−0,11	−0,56
**28.**	**W30s-T attain**	Time of maximum power attainment - W30s	s	7,46	0,91	12,24	0,94	0,83
**29.**	**W30s-T maint**	Time of maximum power maintenance - W30s	s	4,48	0,92	20,47	−0,15	−0,50
**30.**	**VO_2_max_Arm_**	Maximal oxygen uptake –arm work	ml/kg/min	36,32	6,64	18,29	−0,32	−0,16
**31.**	**VO_2_AT_Arm_**	Oxygen uptake at anaerobic threshold – arm work	ml/kg/min	24,37	5,52	22,66	−0,26	−0,69
**32.**	**SI**	Spatial intelligence	n	36,17	9,48	26,22	−1,18	0,50
**33.**	**LC**	Locus of control	n	10,53	4,32	40,97	0,35	0,16
**34.**	**OSB-N**	Neuroticism – raw values	n	6,13	3,90	63,64	0,45	−0,43
**35.**	**OSB-E**	Extroversion – raw values	n	14,60	5,03	34,47	−0,46	−0,44
**36.**	**OSB-P**	Psychotism – raw values	n	10,70	4,18	39,09	−0,28	−0,15
**37.**	**OSB-L**	Lying – raw values	n	8,87	3,31	37,35	0,65	0,40
**38.**	**TEMP-BR**	Briskness – raw values	n	16,43	2,76	16,82	−0,50	−0,44
**39.**	**TEMP-PE**	Perseverance – raw values	n	10,33	4,40	42,56	−0,09	−0,46
**40.**	**TEMP-SS**	Sensory sensitivity – raw values	n	13,27	4,39	33,07	−0,61	−0,06
**41.**	**TEMP-ER**	Emotional reactivity – raw values	n	6,93	4,37	63,06	0,20	−1,01
**42.**	**TEMP-ME**	Mental endurance – raw values	n	12,57	4,99	39,68	−0,83	−0,39
**43.**	**TEMP-AC**	Activity – raw values	n	11,83	3,85	32,49	−0,21	−0,95
**44.**	**Tactics**	Climbing tactics	%	88,37	7,47	8,45	−0,31	−0,54
**45.**	**Technique**	Climbing technique	n	51,07	3,01	5,90	0,22	−0,12

**Table 2:** The results of canonical analysis and the chi-square test (30n)

			Left	Right		
Number of variables			2	7		
Extracted variance			100.00%	32.03%		
Total redundancy			80,.57%	20.76%		
Variables: 1			Max OS	Ape index		
2			Max RP	CRT - errors		
3				Finger strength		
4				E70%z10/10		
5				VO_2_AT_Arm_		
6				TEMP-ME		
7				Technique		
	Rc	Rc^2^	χ^2^	df	p	λ
0	0.935	0.875	131.186	14	0.000	0.088
1	0.542	0.294	18.863	6	0.004	0.705

Canonical R: 0.93546 χ^2^ (14)=131.19 p=0.0000

**Table 3:** The results of canonical analysis for selected mental, technical and physical characteristics with respect to the dependent variables Max OS and Max RP

	**Mental characteristics**		**Technical characteristics**		**Physical characteristics**	

	Canonical R: 0.815 Chi2(16)=73.130 p=0.000		Canonical R: 0.812 Chi2(16)=82.033 p=0.000		Canonical R: 0.815 Chi2(16)=73.130 p=0.000	

	Left	Right	Left	Right	Left	Right
Variance	100.00%	27.84%	100.00%	26.15%	100.00%	37.55%
C. redund.	40.77%	10.85%	52.89%	11.98%	61.81%	20.37%
1	Max OS	LC	Max OS	CRT-errors	Max OS	Mass
2	Max RP	OSB-N	Max RP	Stereometry	Max RP	Ape index
3		OSB-P		Balance-inst		FM%
4		TEMP-BR		Balance-lc		Hip flexion
5		TEMP-PE		Adapt-error		Finger strength
6		TEMP-SS		Adapt-rate		E70%z10/10
7		TEMP-ME		Different		Arm strength
8		Tactics		Technique		VO_2_AT*_Arm_*

**Table 4:** The results of canonical analysis showing correlations between the vectors of the sets of mental, technical and physical characteristics.

	**Technical and physical characteristics**		**Mental and physical characteristics**		**Mental and technical characteristics**	

	Canonical R: 0.815 Chi2(64)=170.42 p=0.000		Canonical R: 0.829 Chi2(64)=146.44 p=0.000		Canonical R: 0.815 Chi2(64)=193.27 p=0.000	

	Left	Right	Left	Right	Left	Right
Variance	100.00%	100.00%	100.00%	100.00%	100.00%	100.00%
C. redund.	**31.80%**	**29.18%**	**30.33%**	**25.28%**	**37.80%**	**38,18%**
1	CRT-errors	Mass	LC	Mass	LC	CRT-errors
2	Stereometry	Ape index	OSB-N	Ape index	OSB-N	Stereometry
3	Balance-inst	FM%	OSB-P	FM%	OSB-P	Balance-inst
4	Balance-lc	Hip flexion	TEMP-BR	Hip flexion	TEMP-BR	Balance-lc
5	Adapt-error	Finger strength	TEMP-PE	Finger strength	TEMP-PE	Adapt-error
6	Adapt-rate	E70%z10/10	TEMP-SS	E70%z10/10	TEMP-SS	Adapt-rate
7	Different	Arm strength	TEMP-ME	Arm strength	TEMP-ME	Different
8	Technique	VO_2_AT*_Arm_*	Tactics	VO_2_AT*_Arm_*	Tactics	Technique
